# Effect of Laboratory Mental Stressors on Cardiovascular Reactivity in Young Women During Different Phases of Menstrual Cycle: An Observational Study

**DOI:** 10.1089/whr.2021.0052

**Published:** 2021-10-21

**Authors:** Aparna Menon, Manisha Kar, Suravi Patra, Sushil Chandra Mahapatra

**Affiliations:** ^1^ Department of Physiology, Dhanalakshmi Srinivasan Medical College and Hospital, Perambalur, India.; ^2^ Department of Physiology and AIIMS Bhubaneswar, Bhubaneswar, India.; ^3^ Department of Psychiatry, AIIMS Bhubaneswar, Bhubaneswar, India.

**Keywords:** heart rate variability, cognitive task, menstrual cycle, mental stress

## Abstract

***Introduction:*** Excessive cardiovascular reactivity to mental stress may be a risk factor for cardiovascular disease. However, there is inconsistent report in the literature regarding change in cardiac autonomic tone with the phase of the menstrual cycle and how it is affected by mental stress. Therefore, the present study was aimed at determining the cardiovascular reactivity to different laboratory mental stressors during follicular and luteal phase of menstrual cycle using heart rate variability (HRV).

***Methods:*** Thirty-three regularly cycling young females (19–35 years of age) were exposed to four cognitive tasks (Stroop test, Mental Rotation test, n-back test, and Mental Arithmetic Stress Test [MAST]) employed as laboratory mental stressors. HRV of the study participants were recorded before, during, and after each cognitive task and the recording was done in both phases of menstrual cycle for each individual.

***Results:*** A significant difference was observed in time domain parameters and nonlinear parameters of HRV in pretest versus during-test condition and during-test versus post-test conditions, but not in frequency domain parameters. No phase difference was found in time domain or frequency domain analysis of HRV in baseline or during performance of task. MAST performance (score out of 50) was significantly higher in luteal than follicular phase, while other tests showed no such difference.

***Conclusion:*** All four mental stress tasks used in the present study were able to elicit significant decrease in parasympathetic tone during performance of task as compared with baseline values of HRV. The present study did not elicit any phase difference in cardiovascular reactivity.

## Introduction

Work stress is a predictor of coronary heart disease (CHD) among men and women.^1–3^ The progression of CHD is reportedly around 10 years delayed in women compared with men because of unique female hormone milieu.^4^ However, as more and more women are joining workforce these days, work stress in women is also evident, which translates into increased incidence of CHD among women.^5^

The parasympathetic limb of autonomic nervous system plays a more dominant role in modulation of the functioning of heart in a very precise and sensitive way. The reactivity of heart to different environmental stimuli depends on bidirectional feedback system between cortical centers regulating vagal activity and vagal feedback system to cortex. This allows improved social and behavioral responses, which could be predicted by cardiac vagal tone as explained by Polyvagal Theory.^6^ Cardiac vagal tank theory suggests that the cardiac vagal activity at stress could be an indicator of how efficiently the responses to stress are mobilized and utilised.^7^ It is evident that the forebrain areas involved in emotion and stress response have neural connections with the autonomic neurons, which innervate the heart.^8–10^

It is emphasized by the neurovisceral integration theory, which postulates that heart rate variability (HRV) may serve as peripheral index of brain areas, involved in execution of cognitive functions such as attention, working memory because of structural and network similarities.^11^ These forebrain areas also show sexually dimorphic activity in terms of expression of sex steroid receptors, proving significant modulatory role played by estrogen and testosterone in cardiac autonomic response to stress.^12^ In young women, a higher reactivity of parasympathetic system was reported as compared with males on exposure to acute stress.^13^ Autonomic response to stress in different phases of the menstrual cycle has been a field of research for a long while. Even then, the evidence from various studies does not suggest any conclusive idea on the change in autonomic reactivity as measured by heart rate or blood pressure response to acute stress.^14^

HRV is a measure of cardiac autonomic tone. The change in resting HRV in response to stressor and its recovery values after stress depend on the individual's self-regulatory mechanism, the environment, or the type of stressor.^7^ The calculation of HRV can be done from electrocardiogram (ECG) recording based on time domain, frequency domain, and nonlinear parameters. It was demonstrated that standard deviation of normal R-R intervals (SDNN), a time-domain parameter of HRV can predict mortality after acute myocardial infarction.^15^ Since then, time-domain parameters have been actively used in research and shown to be a predictor of cardiovascular injury. RMSSD and PNN50 are two other time-domain parameters that indicate cardiac vagal tone.^16^

A high-frequency (HF) component of spectral analysis and SD1 (standard deviation of Poincare plot perpendicular to the line of identity) of nonlinear analysis also indicate parasympathetic control of heart through vagal efferent activity. It was documented that HF component and SD1 are slightly higher in follicular phase than in menstrual phase^17,18^ and luteal phase is associated with an increase in low-frequency (LF) component (a marker of sympathetic modulation, when expressed in normalized unit; or marker of the activity of both sympathetic and vagal limbs) in LF/HF ratio (marker of sympathovagal balance).^19–21^ It suggests increased sympathetic activity during the luteal phase. But other studies have documented higher sympathetic activity in the follicular phase,^22^ and few others have found no phase difference.^23,24^ Furthermore, it was reported that the sympathetic dominance (as evidenced from spectral analysis of HRV) persists in the luteal phase in the presence of mental stressors, although frequency domain and nonlinear parameters of HRV were not examined.^25^

It is understandable that there is inconsistency in the previous reports, which has shown a lacuna in the understanding of changes in cardiovascular reactivity in response to mental stressors in phases of menstrual cycle. So, the present study was aimed at determining the cardiac autonomic tone and cardiovascular reactivity to different laboratory mental stressors during follicular and luteal phase of menstrual cycle using power spectral density, LF/HF ratio, and nonlinear and time-domain analysis of HRV. In the present study, four cognitive tests (Stroop task, mental rotation task, n-back test, and Mental Arithmetic Stress Test [MAST]), which assess various aspects of executive functions, such as inhibitory control, working memory, selective attention, cognitive flexibility, and fluid intelligence, were administered as laboratory mental stressors.

## Materials and Methods

It was a cross-sectional, observational study. The Medical students of 2nd and 3rd year and a few junior and senior residents (19–35 years of age), having stable menstrual cycle (28–32 days) for last 2 months, without any existing systemic disorders were recruited in the present study. The ethical clearance for this study was obtained from the Institutional Ethics Committee (IEC). The study commenced in September 2018 and recruitment of subjects and data collection were completed by January 2020. The recruited subjects were asked to visit Clinical Physiology Laboratory in between days 10–12 of the follicular phase and days 22–25 of the luteal phase, respectively. The respective phases were calculated from expected days of ovulation based on their onset of menstrual cycle and the expected cycle duration. No hormonal or other physiological parameters such as basal body temperature was recorded for the calculation of the different phases of menstrual cycle. Hence, the criteria for the volunteer participants included a narrow range for duration of menstrual cycle (28–32 days) to ensure that the actual phases matched with the calculated days. The sample size was calculated on the basis of an earlier study (comparing LF/HF ratio value in follicular and luteal phase) considering a global statistical power (1-β) of 0.90, level of significance 0.05%, and 10% dropout.^21^ A total of 40 subjects were recruited in this process. Of the 40 recruited participants, two subjects were found to have irregular menstrual cycles following recruitment, and 5 participants were unavailable for a second recording. Therefore, the data from 33 subjects were analyzed.

### Procedures

**FIG. 1.**
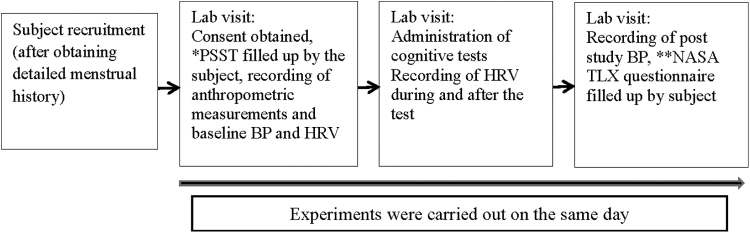
Study flow showing the research protocol followed in the present study.

The study participants were requested to refrain from tea or coffee at least 2 hours before laboratory sessions. Once recruited, the participants were informed of the study protocol in detail, and written consent was obtained from them in the prescribed format as approved by the IEC. Both sessions were conducted, preferably from 5 pm onward, as the serum cortisol level reaches a lower level during that period.^26^ Each session lasted for 45 minutes to 1 hour. The study protocol followed in the study is displayed in Fig. 1.

On arrival at the laboratory, the anthropometric measurements of the subjects were taken. The subjects were asked to fill up Premenstrual Symptom Screening Tool (PSST)^27^ to exclude premenstrual syndrome.

Four cognitive tests were administered to the study participants using computer-based tests developed by Prof. Stoet^28^ namely Stroop task, Mental Rotation test, n back test, and MAST. For Stroop task, the difference in response time for congruent and incongruent answers was taken as the score. Mental rotation test was employed to assess the subject's visuospatial abilities by matching a spatially rotated object with the initial one. N back test was used to assess the working memory of the subject. In the present study, three back test was used, in which alphabets were presented using a custom-made timed presentation. The stimulus was presented for 2 seconds with an interstimulus interval of 2 seconds. A total of 40 such stimuli were presented, and the score was given for correct responses (n) as “n” out of 40. MAST assesses the attention and mathematical aptitude of the subject. A series of autotimed two-digit subtraction were presented on the screen for one second, and the subject received 4 seconds to answer to it verbally. The whole test was presented for a 5-minute duration. The scores were awarded out of 50 for the number of correct responses.

Following cognitive tasks, post-task blood pressure (BP) was measured. Then, National Aeronautics and Space Administration Task Load Index (NASA-TLX) questionnaire was provided to the participants.^29^ NASA-TLX ratings were used as a subjective measurement of mental workload on the tasks.

The subject was asked to visit the laboratory for the next recording on the specified date calculated from the menstrual cycle.

### Recording of HRV parameters

HRV was recorded with PowerLab 4/35 system (AD system, Sydney, Australia) after 5 minutes of rest. The disposable Ag-AgCl surface electrodes were placed on the torso to minimize movement artefact. The baseline BP and HRV of the subjects was recorded following which, four cognitive tasks were applied. For each cognitive task, HRV was recorded before, during, and after each task. The ECG data were digitized at a sampling frequency of 1 KHz. Data were passed through a bandwidth filter in which a low-pass filter was set at 50 Hz, and high-pass filter was set at 0.3 Hz with 50 Hz notch “ON.” The standard time domain, frequency domain, and nonlinear analysis were performed from the recorded HRV.^16,19^

### Statistical analysis

The normality of the data was assessed using Shapiro–Wilk Test. The quantitative data were found to be non-normally distributed and hence comparison between different phases and among pretest, during-test, and post-test values were done using Wilcoxon signed-rank test. The pre, during, and post-test values for physiological parameters (BP, Heart Rate, HRV) were also analyzed using repeated-measures analysis of variance (ANOVA) with two phases being the dependent factor. Sphericity was assessed using Mauchly's test, and Greenhouse–Geisser correction was applied as per the requirement to analyze phase and time-dependent changes in physiological parameters. Bonferroni's *post hoc* test was done to identify which parameters were significantly different in different phases as well as between pre-, during, and post-test. Spearman correlation was applied to examine the association of mood swing, PSST scores, and NASA-TLX scores with the physiological parameters and the test scores for cognitive tests. Statistical analysis was performed using Statistical Package for Social Sciences SPSS 21 version (SPSS, Inc., Chicago, IL). A two-tailed *p*-value <0.05 was taken as the cut-off level of significance.

## Results

Anthropometric measurements of the participants were taken on arrival at the laboratory, and the results are shown in Table 1 below. Among study participants, 10 had body mass index (BMI) more than 23, and 1 person had waist/hip ratio more than 0.81 (cutoff for Asian females).^30^ Two subjects had BMI less than 18 (17.5).

**Table 1.** Basal Characteristics of the Study Participants

	Age (years)	Height (cm)	Weight (kg)	BMI (kg/sq-m)	WHR
*n* = 33	22.2 ± 0.064	157.22 ± 6.093	56.10 ± 9.43	22.71 ± 3.561	0.746 ± 0.064

BMI, body mass index; WHR, waist/hip ratio.

Tables 2–5 display the median values and interquartile range (in parenthesis) of each parameter of HRV before, during, and after the cognitive tests as mentioned in the materials and methods section. There was a significant difference in time domain parameters—heart rate (HR), coefficient of variance between 2 RR intervals (CoVRR), difference in standard deviation of RR intervals (SDSD), standard deviation of adjacent RR intervals (SDRR), percentage of adjacent RR intervals with more than 50 ms difference (PNN50%), root mean square of adjacent RR intervals (RMSSD), and nonlinear parameters—SD1, SD2, SD1/SD2 ratio in pretest versus during test condition and during test versus post-test conditions. However, frequency domain parameters did not show significant change with task performance. Comparison of follicular and luteal phase values did not show significant changes in any of the parameters related to the tests performed. There was no significant change in recorded systolic blood pressure (SBP) and diastolic blood pressure (DBP) and its derivatives such as mean arterial pressure (MAP) and pulse pressure (PP) before and after the task irrespective of the menstrual cycle.

**Table 2.** Heart Rate Variability Parameters Recorded Before, During, and After STROOP Task in Follicular and Luteal Phases of the Menstrual Cycle

STROOP test	Follicular phase			Luteal phase		
HRV parameters	Pretask	During task	Post-task	Pretask	During task	Post-task
Heart rate (bpm)	82.5 (76.7–94.5)^***^^,1,2^	91 (82.8–97.87)	80.09 (76.17–91.1)^***^^,3,2^	87 (78.9–94.2)^***^^,1,2^	89.5 (79.9–97.94)	85.8 (79.27–91.5)^***^^,3,2^
CoVRR	0.071 (0.064–0.09)^**^^,1,2^	0.056 (0.047–0.08)	0.073 (0.062–08)^**^^,3,2^	0.077 (0.06–0.086)^***^^,1,2^	0.057 (0.046–0.078)	0.072 (0.058–0.083)^*^^,3,2^
SDSD (ms)	35.91 (27.55–59.1)^**^^,1,2^	32 (21.32–44.3)	36.69 (28.67–54.4)^***^^,3,2^	38.53 (23.12–55.1)^***^^,1,2^	29.81 (23.19–51.59)	38.39 (22.92–56.09)
SDRR (ms)	53.16 (40.71–70.3)^***^^,1,2^	39.88 (31.75–53.7)	53.69 (43.55–62.1)^***^^,3,2^	56.88 (39.1–63.98)^***^^,1,2^	38.76 (28.98–57.94)	51.59 (41.19–61.62)^**^^,3,2^
PNN50 (%)	15.2 (5.502–39.44)^***^^,1,2^	9.69 (2.26–19.63)	16.67 (8.49–33.52)^***^^,3,2^	15.17 (2.649–34.6)^*^^,1,2^	7.975 (2.158–33.82)	14.04 (4.073–33.07)
RMSSD (ms)	35.87 (29.49–64.95)^***^^,1,2^	31.65 (21.25–44.24)	36.58 (28.4–52.5)^**^^,2,3^	38.49 (23.1–55.1)^**^^,1,2^	29.72 (23.12–51.41)	38.03 (22.86–51.27)
LF nu (ms^2^)	48.91 (36.8–59.22)	45.06 (33.76–52.65)	49.77 (40.26–63.96)	51.35 (34.91–68.39)	43.78 (30.78–53.87)	50.01 (40.7–66.87)
HF nu (ms^2^)	50.23 (38.46–59.32)	50.56 (41.54–60.4)	49.45 (36.90–56.7)	48.17 (30.8–61.97)	49.1 (38.08–61.65)	47.73 (33–56.16)
LF/HF ratio	0.973 (0.0594–1.5)	0.873 (0.477–1.18)	0.982 (0.715–1.72)^*^^,3,2^	1.105 (0.606–2.11)^*^^,1,2^	0.788 (0.537–1.345)	1.02 (0.767–2)^*^^,3,2^
SD1 (ms)	25.39 (20.88–45.96)^***^^,1,2^	22.62 (15.08–28.58)	25.94 (20.27–38.53)^***^^,3,2^	27.24 (16.35–39.91)^**^^,1,2^	21.08 (16.4–36.48)	27.15 (16.21–36.37)
SD2 (ms)	70.25 (54.8–90.21)	52.04 (42.3–70.29)	71.6 (58.92–81.06)	74.42 (45.4–79.77)^**^^,1,2^	49.97 (38.01–73.84)	67.14 (52.22–77.47)^*^^,3,2^
SD1/SD2	0.39 (0.36–0.52)	0.42 (0.34–0.51)	0.4 (0.35–0.52)	0.38 (0.32–0.55)	0.42 (0.37–0.56)	0.42 (0.33–0.49)

The data are displayed as median and interquartile range.

1 Pretask, ^2^task, ^3^post-task.

^*^ *p* < 0.05, ^**^p < 0.01, ^***^p < 0.001.

HF, high frequency; HRV, heart rate variability; LF, low frequency.

**Table 3.** Heart Rate Variability Parameters Recorded Before, During, and After Mental Rotation Task in Follicular and Luteal Phases of Menstrual Cycle

Mental rotation task	Follicular phase			Luteal phase		
HRV parameters	Pretask	During task	Post-task	Pretask	During task	Post-task
Heart rate (bpm)	82 (76.4–89.8)^*^^,1,2^	83.1 (77.8–93.63)	82 (76.6–89.6)^*^^,3,2^	84.9 (76.6–91.8)^***^^,1,2^	87 (81.7–93.6)	85.269 (78.5–85.26)^**^^,3,2^
CoVRR	0.069 (0.06–0.086)^***^^,1,2^	0.059 (0.049–0.068)	0.073 (0.057–0.098)^***^^,3,2^	0.07 (0.059–0.086)^***^^,1,2^	0.061 (0.042–0.068)	0.07 (0.065–0.085)^***^^,3,2^
SDSD (ms)	35.84 (29.55–58.78)^**^^,1,2^	33.73 (25.23–48.74)	38.54 (28.92–64.14)^**^^,3,2^	36.81 (26.47–52.72)^***^^,1,2^	31.09 (19.45–47.99)	38.24 (22.48–50.58)^**^^,3,2^
SDRR (ms)	53.68 (41.15–61.55)^***^^,1,2^	43.72 (32.71–55.04)	49.48 (40.42–62.21)^*^^,3,2^	53.1 (41.97–69.13)^***^^,1,2^	40.47 (28.04–49.43)	52.34 (40.88–63.7)^***^^,3,2^
PNN50 (%)	14.72 (8.654–36.3)^**^^,1,2^	12.5 (5.674–22.58)	18.06 (8.33–31.9)^**^^,3,2^	14.06 (4.72–37.5)^**^^,1,2^	10.18 (2.283–31.79)	14.58 (3.608–32.09)^*^^,3,2^
RMSSD (ms)	35.73 (24.46–58.6)^*^^,1,2^	33.62 (25.18–48.58)	38.36 (28.19–63.92)^**^^,2,3^	36.71 (26.39–52.54)^**^^,1,2^	31 (22.43–47.83)	38.12 (22.42–50.45)^**^^,3,2^
LF nu (ms^2^)	54.71 (36.19–64.79)	48.95 (36.43–64.85)	48.37 (39.01–66.06)	52.15 (40.99–67.09)	55.91 (33.41–61.64)	53.1 (45–70.13)^*^^,3,2^
HF nu (ms^2^)	46.04 (33.71–63.98)	49.38 (34.47–61.71)	52.41 (35.77–60.44)	46.96 (33.64–58.19)	42.89 (34.44–56.75)	46.9 (28.81–55.15)
LF/HF ratio	1.18 (0.565–1.929)	0.973 (0.586–1.881)	1.13 (0.681–2.04)	1.106 (0.705–1.977)	1.263 (0.697–1.681)	1.161 (0.815–2.513)
SD1 (ms)	25.34 (20.9–41.56)^**^^,1,2^	23.85 (17.84–34.46)	27.26 (20.45–45.35)^**^^,3,2^	25.88 (18.19–37.25)^**^^,1,2^	22.19 (15.9–33.93)	27.04 (15.9–35.77)^**^^,3,2^
SD2 (ms)	69.83 (53.87–78.32)	53.55 (42.65–68.83)	74.44 (50.27–92.12)^**^^,2,3^	65.28 (55.23–84.97)^**^^,1,2^	54.46 (35.49–67.1)	67.21 (55.74–81.06)^***^^,3,2^
SD1/SD2	0.41 (0.35–0.5)^*^^,1,2^	0.47 (0.4–0.53)	0.42 (0.32–0.5)	0.4 (0.34–0.53)	0.44 (0.36–0.561)	0.391 (0.29–0.47)^*^^,3,2^

The data are displayed in median and interquartile range.

1 Pretask, ^2^task, ^3^post-task.

^*^ *p* < 0.05, ^**^p < 0.01, ^***^p < 0.001.

**Table 4.** Heart Rate Variability Parameters Recorded Before, During, and After N Back Test in Follicular and Luteal Phases of Menstrual Cycle

N–back test	Follicular phase			Luteal phase		
HRV parameters	Pretask	During task	Post-task	Pretask	During task	Post-task
Heart rate (bpm)	83.9 (77.5–90.2)^***^^,1,2^	90.3 (80.5–96.7)	81.2 (76.9–90.5)^***^^,3,2^	85.63 (78.3–91.5)^***^^,3,2^	89 (82.4–96.99)	84.6 (77.8–90.57)^***^^,3,2^
CoVRR	0.077 (0.062–0.09)^*^^,1,2^	0.064 (0.055–0.079)	0.08 (0.068–0.096)^**^^,3,2^	0.08 (0.056–0.095)^***^^,3,2^	0.06 (0.05–0.076)	0.08 (0.065–0.095)^***^^,3,2^
SDSD (ms)	36.63 (28.46–58.45)^***^^,1,2^	30.38 (24.62–36.05)	37.41 (30.32–37.31)^***^^,3,2^	38.45 (25.58–59.53)^***^^,3,2^	29.48 (19.27–29.48)	41.55 (23.89–49.9)^***^^,3,2^
SDRR (ms)	51.56 (46.02–62.33)^***^^,1,2^	44.16 (34.35–57.34)	57.99 (48.85–72.74)^***^^,3,2^	58.07 (39.72–70.45)^***^^,3,2^	40.29 (32.91–51.93)	58.63 (43.75–70.89)^***^^,3,2^
PNN50 (%)	14.13 (6.41–26.03)^***^^,1,2^	9.843 (3.846–14.352)	15.48 (9.39–35.19)^***^^,3,2^	12.75 (4.583–32.26)^***^^,3,2^	7.477 (1.812–22.22)	18.13 (3.524–31.65)^***^^,3,2^
RMSSD (ms)	34.98 (27.88–42.72)^**^^,1,2^	30.33 (24.58–35.99)	37.31 (30.24–68.56)^***^^,3,2^	38.36 (25.5–59.36)^**^^,1,2^	28.64 (18.22–39.17)	41.41 (23.64–49.75)^***^^,3,2^
LF nu (ms^2^)	62.47 (51.45–68.93)^***^^,1,2^	41.69 (34.21–51.89)	56.08 (46.66–66.92)^*^^,3,2^	55.9 (47.57–72.45)^*^^,1,2^	46.72 (36.69–58.241)	60.02 (52.56–74.28)^**^^,3,2^
HF nu (ms^2^)	36.76 (31.21–45.27)	51.83 (46.17–59.66)	43.46 (32.82–53.27)	43.56 (27.7–49.47)	51.18 (37.31–56.37)	40.76 (26.43–46.58)^*^^,3,2^
LF/HF ratio	1.694 (1.124–2.223)^**^^,1,2^	0.8 (0.566–1.119)	1.29 (0.877–2.035)^*^^,3,2^	1.407 (1.027–2.657)	0.919 (0.673–1.551)	1.473 (1.145–2.818)^**^^,3,2^
SD1 (ms)	25.9 (20.12–41.33)^***^^,1,2^	21.48 (17.41–25.49)	26.45 (21.44–48.62)^***^^,3,2^	27.19 (18.09–60.18)^***^^,1,2^	20.84 (13.63–29.34)	29.38 (16.89–35.28)^***^^,3,2^
SD2 (ms)	66.42 (58.26–85.01)^**^^,1,2^	60.55 (46.15–74.35)	75.93 (63.31–94.3)^**^^,2,3^	75.87 (52.68–74.41)^***^^,1,2^	50.49 (42.01–69.25)	77.54 (59.53–92.78)^***^^,2,3^
SD1/SD2	0.37 (0.31–0.47)	0.37 (0.32–0.42)	0.37 (0.34–0.45)	0.4 (0.3–0.51)	0.41 (0.33–0.49)	0.36 (0.32–0.44)

The data are displayed as median and interquartile range.

1 Pretask, ^2^task, ^3^post-task.

^*^ *p* < 0.05, ^**^p < 0.01, ^***^p < 0.001.

**Table 5.** Heart Rate Variability Parameters Recorded Before, During, and After MAST in Follicular and Luteal Phases of Menstrual Cycle

MAST	Follicular phase			Luteal phase		
HRV parameters	Pretask	During task	Post-task	Pretask	During task	Post-task
Heart rate (bpm)	82.4 (78.8–91)^***^^,1,2^	87.4 (79.51–97)	84 (76.84–91.2)^***^^,3,2^	85.6 (80.4–92.9)^***^^,1,2^	89.1 (83–97.84)	84.8 (79.7–90.6)^***^^,3,2^
CoVRR	0.073 (0.065–0.089)	0.079 (0.067–0.088)	0.081 (0.073–0.097)	0.077 (0.057–0.089)	0.069 (0.056–0.081)	0.081 (0.069–0.094)^**^^,3,2^
SDSD (ms)	34.15 (30.22–5.45)	34.76 (28.65–45.2)	36.81 (29.47–52.42)^*^^,3,2^	36.93 (23.62–56.88)	35.25 (24.29–48.06)	38.9 (26.2–54.48)^**^^,3,2^
SDRR (ms)	53.11 (45.92–63.64)	51.58 (41.87–64.86)	58.74 (48.99–74.33)^**^^,3,2^	56.67 (39.84–65.63)^**^^,1,2^	46.94 (38.56–3.97)	61.45 (47.62–71.5)^***^^,3,2^
PNN50 (%)	15.38 (8.96–25.7)	12.97 (6.051–23.46)	14.56 (8.23–28.24)	17.39 (3.846–31.25)	13.76 (4.665–24.95)	18.33 (6–34.42)
RMSSD (ms)	34.04 (30.1–49.27)	34.73 (28.63–45.13)	36.77 (29.44–52.34)	36.8 (24.17–56.8)^*^^,1,2^	35.21 (24.27–48)	38.86 (26.17–54.4)^*^^,3,2^
LF nu (ms^2^)	61.43 (52.56–72.07)	60.86 (51.07–71.21)	61.03 (51.29–65.71)	61.99 (42.79–73.03)	57 (49.52–60.76)	54.9 (43.14–70.53)
HF nu (ms^2^)	37.58 (27.47–45.71)	38.73 (27.82–47.79)	39.18 (34.36–45.66)	39.93 (27.29–55.63)	41.1 (38.51–48.7)	44.95 (29.84–55.41)
LF/HF ratio	1.845 (1.17–2.625)	1.55 (1.069–2.55)	1.558 (1.11–1.91)	1.55 (0.647–2.67)	1.397 (1.073–1.61)	1.221 (0.778–2.372)
SD1 (ms)	24.14 (21.37–34.95)	24.58 (20.26–31.96)	26.03 (20.84–37.7)	27.37 (17.14–40.22)^**^^,1,2^	24.92 (17.17–33.99)	27.51 (18.53–38.52)^*^^,3,2^
SD2 (ms)	69.58 (60.05–82.83)	69.21 (55.44–84.05)	75.91 (64.84–100.3)^*^^,3,2^	69.17 (53.15–84.75)^*^^,1,2^	61.88 (51.66–78.01)	80.16 (64.72–88.65)^***^^,3,2^
SD1/SD2	0.37 (0.32–0.44)	0.37 (0.28–0.42)	0.34 (0.3–0.43)	0.41 (0.34–0.48)	0.38 (0.35–0.46)	0.35 (0.3–0.43)

The data are displayed as median and interquartile range.

1 Pretask, ^2^task, ^3^post-task.

^*^ *p* < 0.05, ^**^p < 0.01, ^***^p < 0.001.

MAST, Mental Arithmetic Stress Test.

**FIG. 2.**
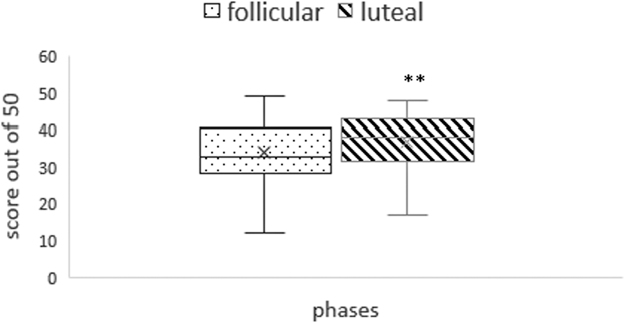
Score of MAST (out of 50) in luteal and follicular phase. *p < 0.01. MAST, Mental Arithmetic Stress Test.

There was no significant change in performance of Stroop task, mental rotation task, or N back test during two phases of the menstrual cycle. However, significant improvement in Mast score was observed during luteal phase as compared with follicular phase (Fig. 2).

PSST questionnaire analysis revealed that 30 participants scored mild and 3 had moderate rating in follicular phase. In the luteal phase, 28 scored mild and 5 had scored moderate rating of premenstrual discomfort. NASA-Tlx questionnaire analysis showed no significant phase-dependent difference of subjective measurement of mental workload on the tasks.

Spearman correlation analysis found no significant correlation between presence of mood swing and HRV parameters, as well as the performance of cognitive tasks.

## Discussion

In the present study, no significant difference was observed between baseline HRV parameters recorded during follicular and luteal phase of the menstrual cycle. The effect size (*r* = 0.02) was found to be too small to extend the inference for larger population. Interestingly, the findings of few previous studies.^23,31^ such as frequency domain parameters, which did not show significant change between phases of menstrual cycle, are in corroboration with the observations made in the present study. In the previous studies, it was noted that sympathetic activity increases as cycle progresses.^20,21^

A significant increase in heart rate of the participant was observed while performing each cognitive task irrespective of the phase of the menstrual cycle. This implies occurrence of definite parasympathetic inactivation associated with sympathetic activation while performing a mental stress test. This finding is in tandem with the neurovisceral integration model as well as the vagal tank theory suggesting a link between the vagal modulation of higher mental functions with that of the vagal modulation of HRV.

Moreover, analysis of time-domain parameters (COVRR, SDSD, SDRR, and pNN50) of HRV revealed a decrease in the value of few time-domain parameters (COVRR, SDSD, SDRR, and pNN50) of HRV during performance of mental stress tasks, which implies inactivation of parasympathetic system and activation of sympathetic system, which corroborate the results of other studies.^32,33^ These changes were phase independent. Frequency analysis of the data exhibited no significant change in LF/HF ratio during Stroop task, mental rotation task, and MAST. However, during N back test, a significant decrease in LF/HF ratio was observed than pre- and post-task values in both phases. This denotes a vagal dominance during task performance, which is contrary to the results otherwise obtained. It is to be reminded that the study participants were in sitting upright posture while recording heart rate, which itself is a contextual variable. In this posture, the primary contributors are PNS activity and baroreflex activity but not sympathetic nervous system (SNS) activity.^34^ Furthermore, nonlinear analysis of HRV also showed no significant phase-dependent changes in SD1, SD2, or SD1/SD2 ratio even though SD1 and SD2 showed significant change during task performance indicative of parasympathetic inactivation and sympathetic activation. It has been reported that the peaks of different ovarian hormones are associated with certain changes in cognitive function as well as cardiac autonomic balance as these hormones selectively modulate brain areas related to autonomic function.^35^ However, the results of the present study showed no phase difference in the cardiac reactivity as measured by HRV between luteal and follicular phase. Few previous studies have also shown similar results.^23^ It was also documented that frequency domain parameters (LF and HF spectral power) were unaltered across menstrual cycle.^31^ Moreover, it can be stated that the computer-based cognitive tests used in the present study (www.psytoolkit.org) have been able to nudge sympathovagal balance of each individual during cognitive tasks and hence are useful tools to be utilized as laboratory mental stressors.

The performance of the study participants for Stroop task, mental rotation task, and n-back test was not significantly different between two phases of the menstrual cycle. In the present study, MAST score achieved by the participants during luteal phase was significantly better than that during the follicular phase. It is a known fact that estrogen and progesterone modulate the expression of other neurotransmitter receptors in the hippocampus and parahippocampal region and thereby fine tune the stress response.^36^ The postovulatory increase in progesterone levels can alter the stress response and cognitive performance in females because of its action on prefrontal cortex.^37^ From the foregoing discussion, it is evident that MAST was more an appropriate mental stressor in terms of significant phase-specific change in performance than any other stressors administered in the present study. MAST was applied for a little longer duration than other three cognitive tests. Therefore, probably, MAST was able to elicit a change in the domains of fluid intelligence tested by it with the change in hormonal milieu, while the other stressors were not able to elicit any such change. Therefore, it warrants the redesigning of the other cognitive tests employed in the present study, although all the four cognitive tests were able to nudge sympathovagal balance in a given subject. Further light could be shed on the issue by doing a comparative analysis of the cognitive tasks across menstrual cycle in a larger sample of the population.

The present study was a within-subject comparison study. All the recordings for the study were obtained after 5 pm so that the serum cortisol levels would be lower and might not interfere with the results of physiological parameters. For each task, HRV was recorded before, during, and after the task. This method was recommended in psychophysiological studies involving HRV to assess the changes produced during performance of task.^7^

The present study has several limitations. In the present study, no hormonal assay was performed to ascertain the menstrual cycle phase of the study participants. The study population was academically high performers. Hence, the results of their performance could not be generalized. Social stress tests like Trier Social Stress Test might provide a better simulation of real-world stressors and their effect on cardiovascular reactivity. Contradictory results regarding basal cardiovascular reactivity in different phases of the menstrual cycle were reported. A larger sample size might be able to throw some light in this context.

## Conclusion

All four mental stress tasks used in the present study were able to elicit significant decrease in parasympathetic tone during performance of task as compared with baseline values as evidenced from change in heart rate and other parameters of HRV. The present study did not elicit any phase difference in cardiac autonomic tone and cardiovascular reactivity in the recruited study participants. Performance of MAST had been found to be significantly better during luteal than follicular phase, but no such difference was observed in the performance of other cognitive tasks.

## Abbreviations Used

ANOVA: analysis of variance
BMI: body mass index
BP: blood pressure
CHD: coronary heart disease
ECG: electrocardiogram
HF: high frequency
HRV: heart rate variability
IEC: Institutional Ethics Committee
LF: low frequency
MAST: Mental Arithmetic Stress Test
NASA-TLX: National Aeronautics and Space Administration Task Load Index
PSST: Premenstrual Symptom Screening Tool
WHR: waist/hip ratio

## References

[1] Schnall PL, Landsbergis PA, Baker D. Job strain and cardiovascular disease. Annu Rev Public Health 1994;15:381–411. 8054091 10.1146/annurev.pu.15.050194.002121

[2] Eaker ED. Psychosocial risk factors for coronary heart disease in women. Cardiol Clin 1998;16:103–111. 9507784 10.1016/s0733-8651(05)70387-8

[3] Cranny CJ, Smith PC, Stone EF. Job satisfaction: How people feel about their jobs and how it affects their performance. New York: Lexington Books, 1992.

[4] Blechman EA, Brownell KD. Behavioural medicine and women: A comprehensive handbook. New York: Gulford Press, 1998.

[5] Czajkowski SM. Psychosocial aspects of women's recovery from heart disease. In: Orth-Gomer K, Chesney M, Wenger NK, eds. Women, stress and heart disease. Mahwah, NJ: Lawrence Erlbaum Associates Publishers, 1998:151–164.

[6] Porges SW. The polyvagal perspective. Biol Psychol 2007;74:116–143. 17049418 10.1016/j.biopsycho.2006.06.009 PMC1868418

[7] Laborde S, Moseley E, Thayer JF. Heart rate variability and cardiac vagal tone in psychophysiological research—recommendations for experiment planning, data analysis and data reporting. Front Psychol 2017;8:2–18. 28265249 10.3389/fpsyg.2017.00213 PMC5316555

[8] Gert J, Horst T Postema F. Forebrain parasympathetic control of heart activity: Retrograde transneuronal viral labeling in rats. Am J Physiol 1997;273:H2926–H2930. 9435633 10.1152/ajpheart.1997.273.6.H2926

[9] Westerhaus MJ, Loewy AD. Central representation of the sympathetic nervous system in the cerebral cortex. Brain Res 2001;903:117–127. 11382395 10.1016/s0006-8993(01)02453-2

[10] Napadow V, Dhond R, Conti G, Makris N, Brown EN, Barbieri R. Brain correlates of autonomic modulation: Combining heart rate variability with fMRI. Neuroimage 2008;42:169–177. 18524629 10.1016/j.neuroimage.2008.04.238 PMC2603289

[11] Thayer JF, Hansen AL, Saus-Rose E, Johnsen BH. Heart rate variability, prefrontal neural function and cognitive performance: The neurovisceral integration perspective on self-regulation, adaptation and health. Ann Behav Med 2009;37:141–153. 19424767 10.1007/s12160-009-9101-z

[12] Kajantie E, Phillips DIW. The effects of sex and hormonal status on the physiological response to acute psychosocial stress. Psychoneuroendocrinology 2006;31:151–178. 16139959 10.1016/j.psyneuen.2005.07.002

[13] Dart AM, Du XJ, Kingwell BA. Gender, sex hormones and autonomic nervous control of the cardiovascular system. Cardiovasc Res 2002;53:678–687. 11861039 10.1016/s0008-6363(01)00508-9

[14] Collins KJ, Easton JC, Belfield-Smith H, Exton-Smith AN, Pluck RA. Effects of age on body temperature and blood pressure in cold environments. Clin Sci 1985;69:465–470. 10.1042/cs0690465 4042547

[15] Kleiger RE, Miller JP, Bigger JT, Moss AJ, The Multicenter Post-Infarction Research Group. Decreased heart rate variability and its association with increased mortality after acute myocardial infarction. Am J Cardiol 1987;59:256–262. 3812275 10.1016/0002-9149(87)90795-8

[16] Shaffer F, Ginsberg JP. An overview of heart rate variability metrics and norms. Front Public Health 2017;5:1–17. 29034226 10.3389/fpubh.2017.00258 PMC5624990

[17] Saeki Y, Atogami F, Takahashi K, Yoshiawa T. Reflex control of autonomic function induced by posture change during the menstrual cycle. J Auton Nerv Syst 1997;66:69–74. 9334995 10.1016/s0165-1838(97)00067-2

[18] Bai X, Li J, Zhou L, Li X. Influence of the menstrual cycle on nonlinear properties of heart rate variability in young women. Am J Physiol Heart Circ Physiol 2009;297:765–774. 10.1152/ajpheart.01283.2008 19465541

[19] Malik M, Bigger JT, Camm AJ, et al. Heart rate variability: Standards of measurement, physiological interpretation and clinical use. Eur Heart J 1996;17:354–381. 8737210

[20] Sato N, Miyake S, Akatsu J, Kumashiro M. Power spectral analysis of heart rate variability in health young women during the normal menstrual cycle. Psychosom Med 1995;57:331–335. 7480562 10.1097/00006842-199507000-00004

[21] Yuki T, Takahiro Y, Yasutake T, et al. The impact of menstrual cycle phases on cardiac autonomic nervous system activity: An observational study considering lifestyle (diet, physical activity, and sleep) among female college students. J Nutri Sci Vitaminol (Tokyo) 2017;63:249–255. 10.3177/jnsv.63.249 28978872

[22] Princi T, Parco S, Accardo A, et al. Parametric evaluation of heart rate variability during the menstrual cycle in young women. Biomed Sci Instrum 2005;41:340–345. 15850129

[23] Leicht AS, Hirning DA, Allen GD. Heart rate variability and endogenous sex hormones during the menstrual cycle in young women. Exp Physiol 2003;88:441–446. 12719769 10.1113/eph8802535

[24] Nakagawa M, Ooie T, Takahashi N, et al. Influence of menstrual cycle on QT interval dynamics. Pacing Clin Electrophysiol 2006;29:607–613. 16784426 10.1111/j.1540-8159.2006.00407.x

[25] Sato N, Miyake S. Cardiovascular reactivity to mental stress: Relationship with menstrual cycle and gender. J Physiol Anthropol Appl Human Sci 2004;23:215–223. 10.2114/jpa.23.215 15599065

[26] Oster H, Challet E, Ott V, et al. The functional and clinical significance of the 24-hour rhythm of circulating glucocorticoids. Endocr Rev 2017;38:3–45. 27749086 10.1210/er.2015-1080 PMC5563520

[27] Steiner M, Macdougall M, Brown E. The Premenstrual Symptoms Screening Tool (PSST) for clinicians. Arch Womens Ment Health 2003;6:203–209. 12920618 10.1007/s00737-003-0018-4

[28] Stoet G. PsyToolkit: A software package for programming psychological experiments using Linux. Behav Res Methods 2010;42:1096–1104. 21139177 10.3758/BRM.42.4.1096

[29] Hart SG, Staveland L. Development of NASA-TLX (Task Load Index): Results of empirical and theoretical Research. Adv Psychol 1988;52:139–183.

[30] Snehalatha C, Viswanathan V, Ramachandran A. Cutoff values for normal anthropometric variables in Asian Indian adults. Diabetes Care 2003;26:1380–1384. 12716792 10.2337/diacare.26.5.1380

[31] Yildirir A, Kabakci G, Akgul E, Tokgozoglu L, Oto A. Effects of menstrual cycle on cardiac autonomic innervation as assessed by heart rate variability. Ann Noninvasive Electrocardiol 2001;7:60–63. 10.1111/j.1542-474X.2001.tb00140.x PMC7027654 11844293

[32] Rajbhandari Pandey K, Khadka R, Agrawal K, Paudel BH, Panday DR. Cardiovascular reactivity to acute mental stress in post ovulatory females. J Nepal Health Res Counc 2021;18:626–631. 33510500 10.33314/jnhrc.v18i4.2555

[33] Simon SG, Sloan RP, Thayer JF, Jamner LD. Taking context to heart: Momentary emotions, menstrual cycle phase, and cardiac autonomic regulation. Psychophysiology 2021;58:e13765. 33453074 10.1111/psyp.13765

[34] Shaffer F, McCraty R, Zerr CL. A healthy heart is not a metronome: An integrative review of the heart's anatomy and heart rate variability. Front Psychol 2014;5:1040. 25324790 10.3389/fpsyg.2014.01040 PMC4179748

[35] Bethea CL, Lu NZ, Gundlah C, Streicher JM. Diverse actions of ovarian steroids in the serotonin neural system. Front Neuroendocrinol 2002;23:41–100. 11906203 10.1006/frne.2001.0225

[36] Oyola MG, Handa RJ. Hypothalamic–pituitary–adrenal and hypothalamic–pituitary–gonadal axes: Sex differences in regulation of stress responsivity. Stress 2017;20:476–494. 28859530 10.1080/10253890.2017.1369523 PMC5815295

[37] Ossewaarde L, Hermans EJ, van Wingen GA, et al. Neural mechanisms underlying changes in stress-sensitivity across the menstrual cycle. Psychoneuroendocrinology 2010;35:47–55. 19758762 10.1016/j.psyneuen.2009.08.011

